# Efficacy and safety of image-guided hypofractionated radiotherapy for hepatocellular carcinoma with portal vein tumor thrombosis: a retrospective, multicenter study

**DOI:** 10.1186/s12885-025-13739-3

**Published:** 2025-04-21

**Authors:** Sang Min Lee, Jin Hwa Choi, Jung-Hwan Yoon, Yoon Jun Kim, Su Jong Yu, Jeong-Hoon Lee, Hyun-Cheol Kang, Eui Kyu Chie, Kyung Su Kim

**Affiliations:** 1 Department of Radiation Oncology, Seoul National University Hospital, Seoul National University College of Medicine, Seoul, Republic of Korea; 2 Department of Radiation Oncology, Chung-Ang University Hospital, Chung-Ang University College of Medicine, Seoul, Republic of Korea; 3 Department of Internal Medicine and Liver Research Institute, Seoul National University Hospital, Seoul National University College of Medicine, Seoul National University, Seoul, Republic of Korea

**Keywords:** Hepatocellular carcinoma, Portal vein tumor thrombosis, Image-guided radiation therapy, Hypofractionated radiation therapy

## Abstract

**Background:**

External beam radiation therapy (RT) has shown promising effects for hepatocellular carcinoma (HCC) patients with portal vein tumor thrombosis (PVTT) in recent studies. However, there is still a lack of consensus on the optimal RT scheme for PVTT treatment. We evaluated the efficacy and safety of image-guided 10-fraction hypofractionated RT in these patients.

**Methods:**

Between January 2016 and March 2022, a total of 95 HCC patients with PVTT received 10-fraction hypofractionated image-guided radiation therapy (IGRT) at two institutes, and 69 patients were analyzed. Follow-up imaging was performed at three-month intervals after the completion of RT. The extent of PVTT was described according to the Liver Cancer Study Group of Japan classification: Vp1 = segmental portal vein branch, Vp2 = right/left anterior/posterior portal vein, Vp3 = right/left portal vein, and Vp4 = main portal vein. Response evaluation was performed using Response Evaluation Criteria in Solid Tumors, version 1.1. Freedom from local progression (FFLP), progression-free survival (PFS), and overall survival (OS) were calculated from the start date of RT.

**Results:**

The median prescribed dose of 50 Gy (range: 40-50 Gy; biologically effective dose [BED]: 56-75Gy_10_) was delivered in 10 fractions. In this cohort, 4.3% of patients had Vp1, 20.3% had Vp2, 37.7% had Vp3, and 37.7% had Vp4. Median Planning target volume was 105.3 cc (interquartile range [IQR], 74.1–179.4 cc). Fifty-two (75.4%) patients received 50 Gy. With a median follow-up of 10.2 months (IQR, 6–21 months), the median OS was 20.3 months, and 1-year FFLP, PFS, and OS rates were 88.7%, 26.9%, and 62.2%, respectively. At 3 months follow-up, 13.0% had a complete response, 36.2% had a partial response, 46.4% had a stable disease and 4.4% had a progressive disease. In the multivariate analysis, alpha-fetoprotein level ≥ 600 IU/ml (hazard ratio [HR] 2.06, *p* = 0.03), Child–Pugh Class B or C (HR 2.30, *p* = 0.02), and stage IVA or IVB (4.05, *p* = 0.02) were significantly related to OS. During the follow-up period, there were 2 (2.9%) cases of grade ≥ 3 toxicity: grade 3 liver enzyme elevation (*n* = 1), and acute cholangitis (*n* = 1).

**Conclusions:**

Hypofractionated RT demonstrated promising local PVTT control and OS rates with acceptable toxicity. These data suggest that 10-fraction image-guided hypofractionated RT (BED: 56–75 Gy_10_) is a feasible treatment option for PVTT in HCC patients.

## Background

Hepatocellular carcinoma (HCC) is the most common histological subtype of primary liver cancer in adults, accounting for 75–85% of all primary liver cancers. It was the third leading cause of cancer-related deaths worldwide in 2020 [1, 2]. Among HCC patients, portal vein tumor thrombosis (PVTT) is a common and significant complication, occurring in 36.8–62.2% of cases [3–7]. The presence of PVTT markedly worsens prognosis, with median survival declining from 35.7 months to 7.2 months, depending on the extent of the thrombosis [8, 9].

The Barcelona Clinic Liver Cancer (BCLC) staging system categorizes PVTT as an advanced stage (stage C), for which systemic therapies such as the atezolizumab-bevacizumab combination or durvalumab-tremelimumab are recommended as first-line treatments [10–12]. Despite significant advancements in systemic therapies, local treatment modalities—such as transarterial chemoembolization (TACE), radioembolization, and radiation therapy (RT)—continue to play a critical role in managing PVTT. This is particularly relevant in regions like East Asia, where the prevalence of HCC and PVTT is higher due to elevated rates of HBV infection [13, 14]. Local treatments can also serve as a bridging therapy, for preserving future systemic treatment options. Among these modalities, RT has the advantage of being feasible even for patients who are unsuitable for TACE or other invasive local treatments.

From the perspective of RT, several fractionation schemes have been explored for PVTT, including conventional fractionation, stereotactic body radiotherapy (SBRT), and hypofractionated RT. Although studies have shown that conventional RT can be effective in treating PVTT, its efficacy appears to be more pronounced in patients receiving a biologically effective dose (BED) of at least 58 Gy_10_ (α/β ratio = 10 Gy) or higher. This would require a dose of at least 50 Gy delivered in 25 fractions (2 Gy per fraction, BED = 60 Gy_10_), resulting in a treatment duration of at least 5 weeks, which could be a drawback considering the rapid disease progression often seen in PVTT patients [15–17]. SBRT, which involves higher doses delivered in 1–5 fractions, has demonstrated promising local control (1-year local control rates of 87–95%) but is associated with higher risks of toxicities, especially when treating tumors adjacent to critical structures [18, 19]. Proton beam therapy (PBT), with its ability to deliver high-dose radiation while sparing surrounding tissues, has shown excellent outcomes in some studies. However, like conventional RT and SBRT, it requires further validation through larger trials [20, 21].

More broadly, studies have indicated that the optimal dose and fractionation for RT in HCC remain unclear. This highlights the need for tailored approaches to balance efficacy and safety across different RT modalities [22–24].

In this study, we evaluated the efficacy and safety of a 10-fraction image-guided hypofractionated RT for HCC patients with PVTT. This moderate-dose approach aims to balance the benefits of hypofractionation with minimized toxicity and may provide an effective option to bridge patients to modern systemic therapy.

## Patients and methods

### Patients

This study was a retrospective, multicenter investigation conducted at Seoul National University Hospital and Chung-Ang University Hospital. Eligible patients for this study were those diagnosed with primary HCC, with radiologically confirmed PVTT, who received 10 fractions of image-guided hypofractionated RT. Patients were required to be 20 years of age or older, have an Eastern Cooperative Oncology Group (ECOG) performance status of 2 or less, and a Child–Pugh class of A or B. Only patients with a follow-up period of 3 months or more were included. Additionally, patients needed to have no evidence of extrahepatic metastasis, as confirmed by abdominal CT, chest CT, and/or PET-CT scans. Patients who did not complete the entire RT course or received a dose of less than 40 Gy were excluded. The diagnosis of HCC was based on radiologic findings from computed tomography (CT) or magnetic resonance imaging (MRI) and elevated serum alpha-fetoprotein (AFP) concentrations (≥ 200 IU/ml), following the guidelines of the Korean Liver Cancer Study Group and the National Cancer Center [25].

PVTT was detected based on the occurrence of a low-attenuation intraluminal filling defect during the portal phase or an enhancement in the filling defect during the arterial phase. The extent of PVTT was described according to the Liver Cancer Study Group of Japan classification: Vp0 = no PVTT, Vp1 = presence of a PVTT distal to the second-order portal vein branches, Vp2 = presence of a PVTT in the second-order portal vein branches, Vp3 = presence of a PVTT in the first-order portal vein branches, and Vp4 = presence of a PVTT in the main trunk of the portal vein or a portal vein branch contralateral to the primarily involved lobe (or both) [26].

HCCs were classified according to the modified Union for International Cancer Control (mUICC) staging system proposed by the Liver cancer study group of Japan [27, 28].

This study received approval from the institutional review board of the participating institutes, and written informed consent was waived due to the retrospective nature of the study. (IRB No. H-2205–143-1327).

### Treatment

Patients were in a supine position with both arms raised above the head for a 10-phase four-dimensional-CT simulation. Free breathing with abdominal compression via a plate was applied during the CT scan to minimize liver movement [29]. The information from the CT scan was then inputted into the Eclipse planning system (Varian, Palo Alto, CA).

The gross tumor volume (GTV) of PVTT was contoured on the portal phase of liver CT. The decision to include or exclude intrahepatic tumors in the GTV was made by the radiation oncologist, considering the tumor size, patient's liver function, and the expected irradiation volume of the liver. The internal target volume (ITV) was defined as the summation of the GTVs on all the 10 phases of CT images, and the planning target volume (PTV) was expanded from the ITV with a 0.5–0.6 cm margin [30]. The normal liver, stomach, duodenum, and small bowel were contoured as organs-at-risk (OARs).

RT was delivered using volumetric-modulated radiotherapy (VMAT) with a 6 MV output linear accelerator. A dose of 50 Gy in 10 fractions over 2 weeks was delivered to the target volume. Although the aim was to deliver 50 Gy/10fx to the 95% isodose line of the PTV, the dose was reduced to 40 Gy/10fx when the dose constraints of the OARs were a concern. Even in cases treated with 50 Gy/10fx, some parts of the PTV were treated with a lower dose through a simultaneous integrated boost (SIB), considering the proximity to OARs and dose fall-off. In such cases, we aimed to deliver at least 40 Gy/10fx to the 95% isodose line of the PTV.

To meet dose constraints for OARs, dose volumetric parameters were calculated using the dose-volume histogram (DVH). The mean liver dose and/or D_700cc_ (D_n_, the minimal doses received by the highest irradiated volumes, n) of the liver were kept below 24 Gy, respectively. D_0.03 cc_ of the stomach was kept below 45 Gy, and the D_50cc_ was below 33.9 Gy. For the duodenum, the D_0.03 cc_ was kept below 45 Gy, and the D_5cc_ was below 33.9 Gy. Additionally, the D_0.03 cc_ for the small bowel was kept below 41 Gy [30–32].

At each fraction, IGRT via cone-beam CT was used to position the patient. Any discrepancies between the target lesion and organs at risk (OARs) from their initial positions on the simulation CT were manually adjusted along three axes (longitudinal, vertical, and lateral).

### Treatment and toxicity evaluation

After completion of treatment, patients were followed up every 2–3 months for the first 12 months, every 6 months up to 36 months, and then yearly for up to 5 years or until death. Blood samples and three-phase dynamic liver CT or MRI were taken at each follow-up. Tumor response was assessed using Response Evaluation Criteria in Solid Tumors (RECIST) version 1.1. Toxicity was graded based on the National Cancer Institute Common Terminology Criteria for Adverse Events (CTCAE) version 5.0.

### Statistics

The primary outcome, overall survival (OS), was defined as the time from the first day of RT to the date of death from any cause. Secondary outcomes included freedom from local progression (FFLP), defined as the time to in-field disease progression or death from any cause; progression-free survival (PFS), defined as the time to intrahepatic in-field and/or out-field disease progression or death from any cause; and distant metastasis-free survival (DMFS), defined as the time to the appearance of distant metastasis or death. Additional secondary outcomes were the response rate of PVTT, acute toxicity, and late toxicity of OARs.

The cumulative incidence for local recurrence with the competing risk of death without local recurrence was estimated with the Fine-Gray analysis. The FFLP was calculated based on the cumulative incidence. The PFS, DMFS, and OS rates were estimated using the Kaplan–Meier method. Factors influencing these survival rates were identified through Cox's regression analysis, wherein significant variables (*p* < 0.2) from univariate analysis and clinically important variables were incorporated into a multivariate analysis. The Wilcoxon signed-rank test was used to compare liver function before and after treatment. R software v. 4.4.1 and Stata 17.0 was used for calculations. A *p*-value of < 0.05 was considered statistically significant.

## Results

### Baseline patient characteristics

**Fig. 1:**
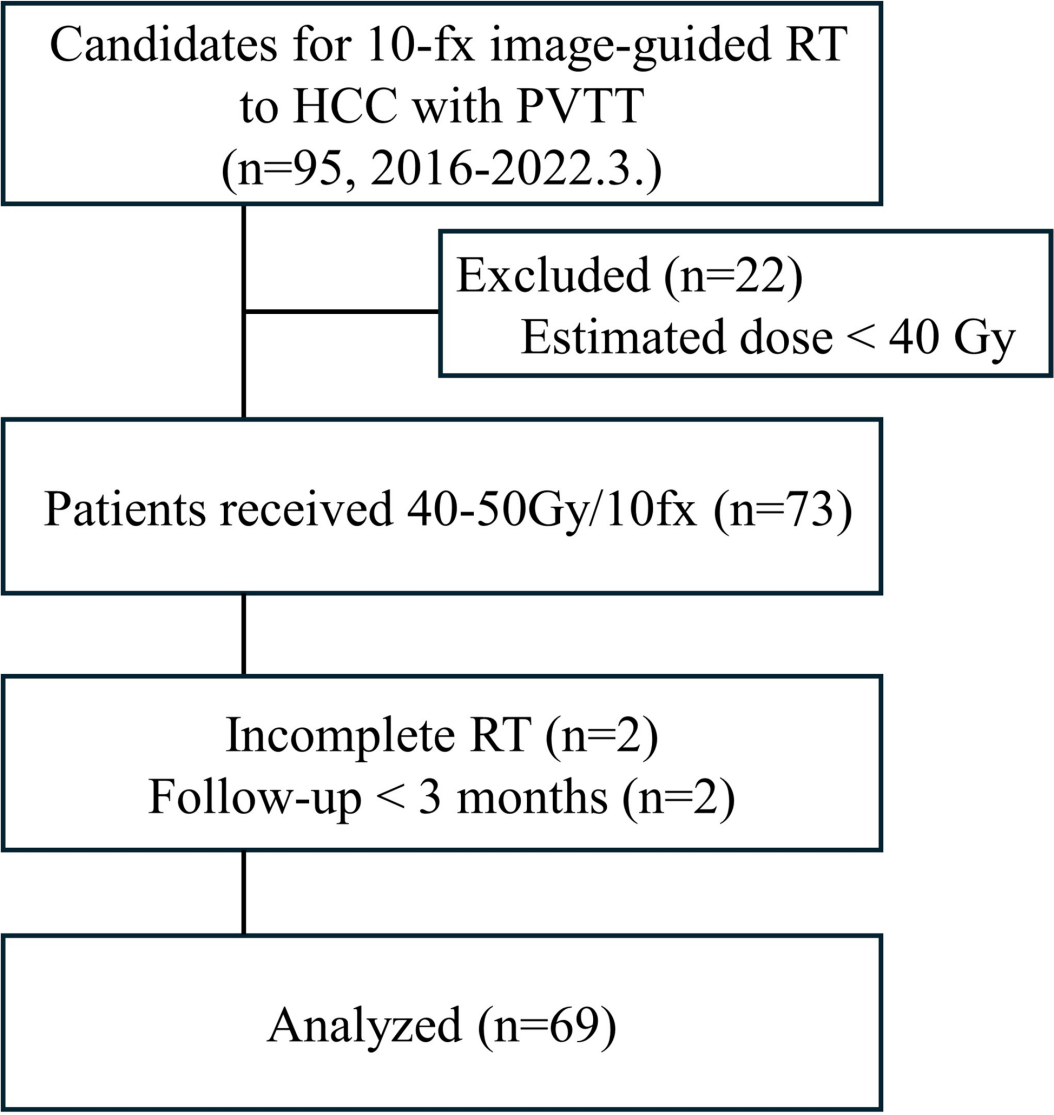
CONSORT diagram of retrospective cohort. fx, fraction; RT, radiation therapy; HCC, hepatocellular carcinoma; PVTT, portal vein tumor thrombosis

Between January 2016 and March 2022, a total of 95 HCC patients with PVTT received 10-fraction hypofractionated image-guided radiation therapy (IGRT) at two institutes, and 69 patients were analyzed (Fig. 1). Patient characteristics are presented in Table 1. The mean age of the patients was 63 years (range: 31–84). Many of the patients (88.4%) were male. Most of the patients (89.9%) had a good performance status (ECOG 0 or 1), and the majority (76.8%) had Child–Pugh A liver function at the time of RT. Chronic viral hepatitis B or C infection was present in most of the patients (82.6%). The median baseline AFP and protein induced by vitamin K absence-II (PIVKA-II) levels were 188 IU/ml (IQR, 13.2–4059) and 340 mAU/ml (IQR, 69–4218), respectively. PVTT involved the main trunk or bilateral first branch of portal vein (Vp4) in 26 patients (37.7%), the unilateral first branch of portal vein (Vp3) in 26 patients (37.7%), and the second branch of portal vein (Vp2) in 14 patients (20.3%).

**Table 1:** Patient characteristics

Variable	n	(%)
**Age (years)**	63	(range, 31–84)
**Sex**		
Female	8	(11.6)
Male	61	(88.4)
**ECOG**		
0	22	(31.9)
1	40	(58.0)
2	7	(10.1)
**Child–Pugh Class**		
A	53	(76.8)
B	16	(23.2)
**Underlying liver disease**		
Hepatitis B	49	(71.0)
Hepatitis C	8	(11.6)
Alcohol related	4	(5.8)
Others^a^	4	(5.8)
None	4	(5.8)
**Baseline AFP (IU/ml), median**	188	(IQR, 13.2–4059)
**Baseline PIVKA-II (mAU/ml), median**	340	(IQR, 69–4218)
**mUICC Stage**		
I	0	(0.0)
II	3	(4.4)
III	17	(24.6)
IVA	45	(65.2)
IVB	4	(5.8)
**Previous treatments**	69	(100)
Surgery	8	(11.6)
TACE	69	(100)
RFA / PEIT	15	(21.7)
TARE	1	(1.4)
Sorafenib/Lenvatinib	6	(8.7)
**Multiple tumor**	42	(60.9)
**PVTT location**^b^		
Vp1	3	(4.3)
Vp2	14	(20.3)
Vp3	26	(37.7)
Vp4	26	(37.7)
**PTV volume (cc), median**	105.3	(IQR, 74.1–179.4)
**BED**_**10**_** (Gy), median**	75	(range, 56–75)

Abbreviations: *ECOG* Eastern Cooperative Oncology Group, *mUICC* Modified Union for International Cancer Control Stage, *AFP* Alpha-Fetoprotein, *PIVKA-II* Protein induced by vitamin K absence or antagonist II, *IQR* Interquartile range, *TACE* Trans arterial chemoembolization, *RFA* Radiofrequency ablation, *PEIT* Percutaneous ethanol injection therapy, *TARE* Trans arterial radioembolization, *PVTT* Portal vein tumor thrombosis, *PTV* Planning target volume, *BED*_*10*_ Biologically effective dose, as the α/β = 10

^a^Others: Non-alcoholic fatty liver disease, Toxic hepatitis

^b^Liver Cancer Study Group of Japan classification: Vp0 = no PVTT, Vp1 = presence of a PVTT distal to, but not in, the second-order portal vein branches, Vp2 = presence of a PVTT in the second-order portal vein branches, Vp3 = presence of a PVTT in the first-order portal vein branches, and Vp4 = presence of a PVTT in the main trunk of the portal vein or a portal vein branch contralateral to the primarily involved lobe (or both)

Before receiving RT for PVTT, all patients had undergone treatments other than RT for HCC. These treatments include both those for PVTT and HCC without PVTT. Specifically, all patients had previously undergone TACE, 15 patients (21.7%) received radiofrequency ablation (RFA) or percutaneous ethanol injection therapy (PEIT), 8 patients (11.6%) received hepatectomy, and 6 patients (8.7%) received Sorafenib or Lenvatinib.

At the time of RT, 42 patients (60.9%) had 2 or more discrete tumors, including PVTT, within the liver. According to the mUICC staging system, 45 patients (65.2%) were in stage IVA, and 17 patients (24.6%) were in stage III. The median PTV volume was 105.3 cc (IQR, 74.1–179.4). The median BED with an α/β ratio of 10 was 75 Gy_10_ (range: 56–75 Gy_10_).

Following RT, 18 patients (26.1%) received TACE, 22 patients (31.9%) underwent systemic therapy, 2 patients (2.9%) received both TACE and systemic therapy, and 27 patients (39.1%) did not receive any further treatment. The median time to subsequent treatment was 1.2 months (IQR, 0.6–3.2).

### Response rate

The radiologic response of PVTT at 3 months, 6 months, 9 months, and 12 months of follow-up was summarized in Table 2. At the 3-month follow-up, all patients were evaluated, and the responses were as follows: complete response (CR) in 9 (13.0%) patients, partial response (PR) in 25 (36.2%) patients, stable disease (SD) in 32 (46.4%) patients, and progressive disease (PD) in 3 (4.4%) patients. The response rate (CR + PR) of the PVTT was 49.2%. At the 6-month, 9-month, and 12-month follow-ups, although not all patients were investigated, the response rates were 60.0%, 63.9%, and 68.9%, respectively.

**Table 2:** Response rate

Response^a^	3 months		6 months		9 months		12 months	
CR	9	(13.0%)	23	(46.0%)	21	(58.3%)	17	(58.6%)
PR	25	(36.2%)	7	(14.0%)	2	(5.6%)	3	(10.3%)
SD	32	(46.4%)	15	(30.0%)	12	(33.3%)	7	(24.1%)
PD	3	(4.4%)	5	(10.0%)	1	(2.8%)	2	(6.9%)
Total	69		50		36		29	

^a^Tumor response was evaluated according to The Response Evaluation Criteria in Solid Tumors, version 1.1; CR, complete response; PR, partial response; SD, stable disease; PD, progressive disease

### Freedom from local progression, Progression-free survival, and distant metastasis-free survival

**Fig. 2:**
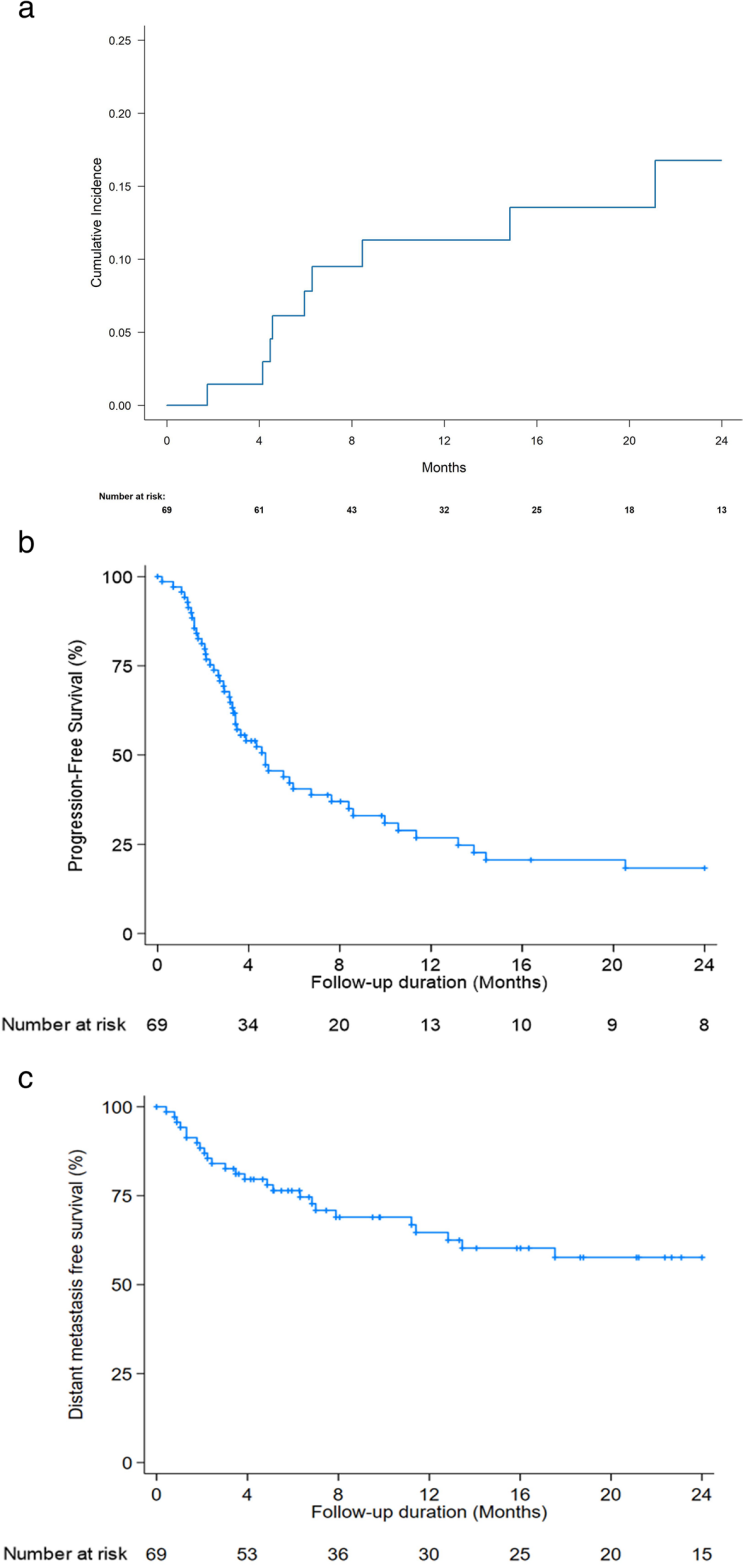
Cumulative local recurrence rate (**a**), Progression fee survival (**b**) and Distant metastasis free survival (**c**), respectively

The median follow-up duration was 10.2 months (IQR, 6–21), and at the time of analysis, 29 patients were alive, and 40 had died from disease progression. Out of 69 patients, disease progression occurred in 60 (87.0%), and the patterns of disease progression were as follows: in-field progression in 9 (13.0%) patients, out-field intrahepatic progression in 52 (75.4%), and distant metastasis in 30 (43.5%). Among cases of in-field recurrence (*n* = 9), tumor regrowth was observed as diffuse enlargement rather than focal progression near specific regions or OARs. The median times to in-field, intrahepatic in-field and/or out-field progression, and distant metastasis were 9.8 months (range: 1.6–65.7), 4.7 months (95% CI, 3.2–7.6), and 30.6 months (95% CI, 12.8–47.9), respectively. The 1-year FFLP, PFS, and DMFS were 88.7%, 26.9%, and 64.7%, respectively (Fig. 2).

### Overall survival and related factors

**Fig. 3:**
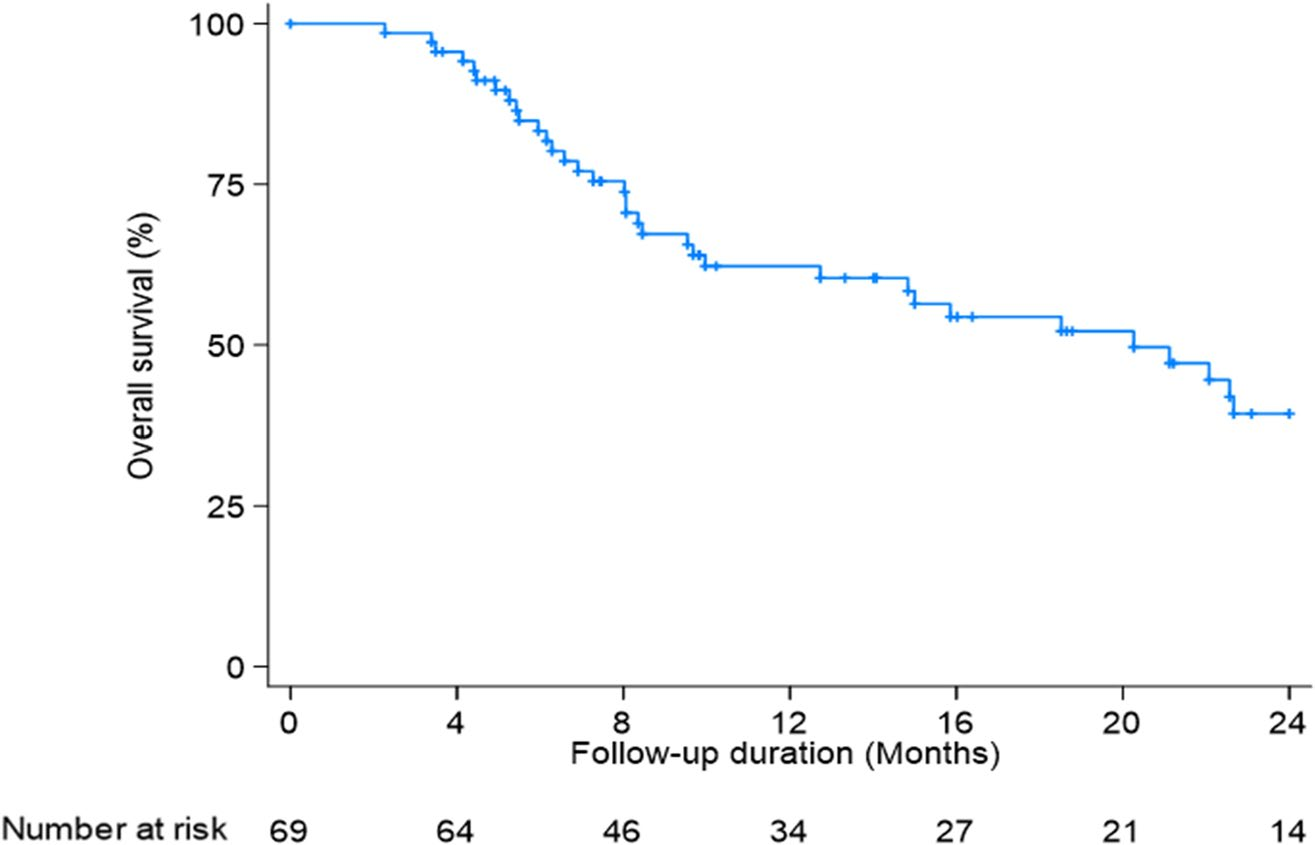
Kaplan–Meier curve of overall survival

**Fig. 4:**
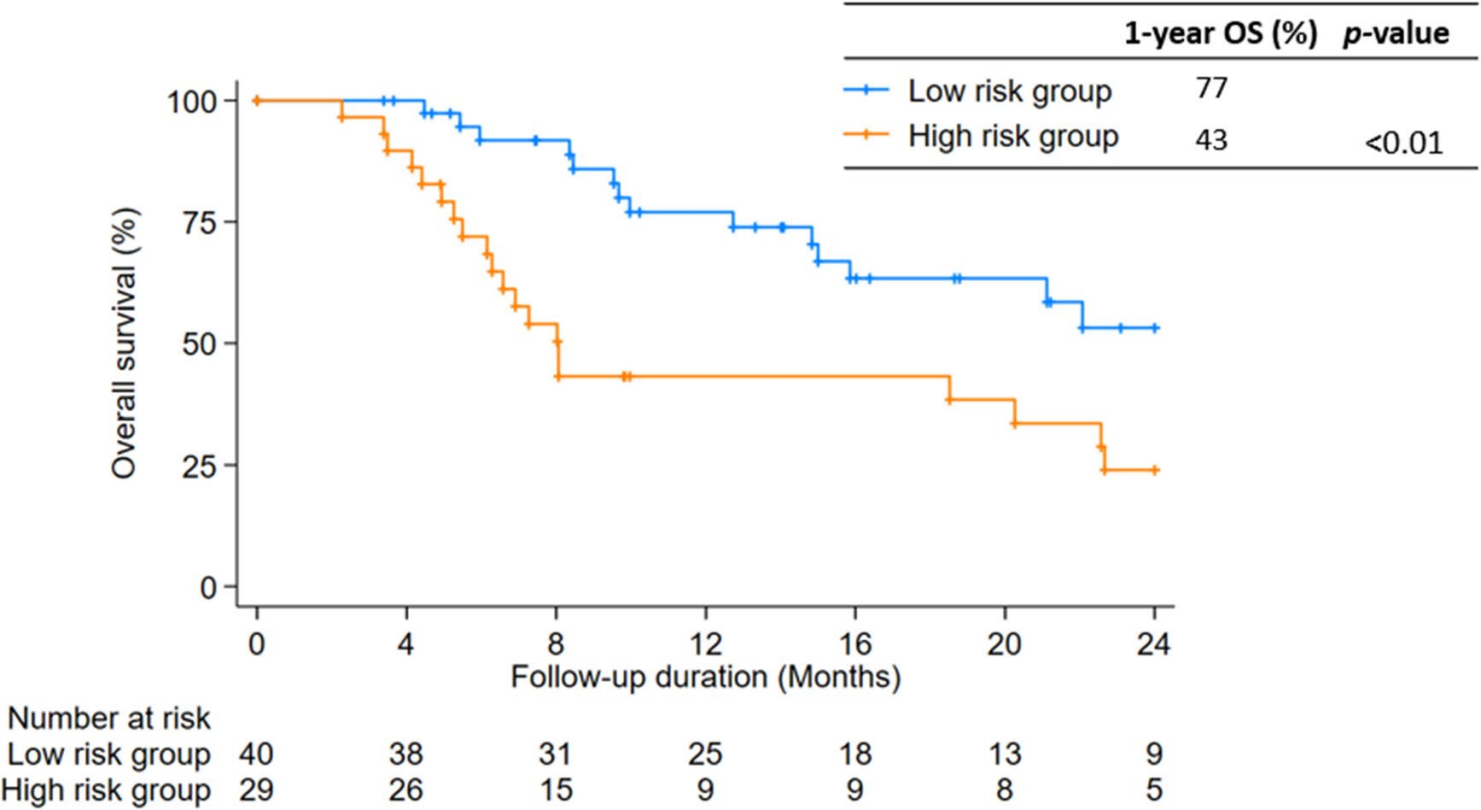
Patients were divided in 2 groups according to the number of risk factors. The Kaplan–Meier curve of OS between two groups are shown. Risk factors are mUICC stage (IVA or IVB), AFP ≥ 600 IU/ml and Child–Pugh Class B or C. Low risk group = 0–1 risk factors. High risk group = 2–3 risk factors. OS, overall survival; mUICC stage, modified Union for International Cancer Control stage; AFP, Alpha-Fetoprotein

The overall survival rate at 1 year was 62.2% with a median survival of 20.3 months (IQR, 8.0–35.2) (Fig. 3). In the multivariate analysis, baseline AFP, baseline Child–Pugh class, and mUICC stage were significant predictors of OS (Table 3). Patients with baseline AFP ≥ 600 IU/ml, Child–Pugh class B or C, and mUICC stage IVA or IVB had a worse prognosis, with HR of 2.06 (95% CI, 1.06–4.01, *p* = 0.03), HR 2.30 (95% CI, 1.12–4.75, *p* = 0.02), and HR 4.05 (95% CI, 1.29–12.74, *p* = 0.02), respectively. Additionally, patients who had 1 or none of the three significant predictors for OS showed better 1-year OS compared to patients who had 2 or 3 (77% vs 43%, *p* < 0.01) (Fig. 4).

**Table 3:** Multivariate analysis of prognostic factors for overall survival

Variable	HR	(95% CI)	*p*-value
PVTT at Vp4^a^	0.72	(0.35—1.51)	0.39
PR or CR at 3 months	0.86	(0.42—1.78)	0.88
PTV volume ≥ 170 cc	1.77	(0.84—3.74)	0.13
AFP ≥ 600 IU/ml	2.06	(1.06—4.01)	0.03
Child–Pugh Class B or C	2.30	(1.12—4.75)	0.02
mUICC stage IVA or IVB	4.05	(1.29—12.74)	0.02

Abbreviations: *PVTT* Portal vein tumor thrombosis, *CR* Complete response, *PR* Partial response, *PTV* Planning target volume, *AFP* Alpha-Fetoprotein, *mUICC* Modified Union for International Cancer Control Stage

^a^Liver Cancer Study Group of Japan classification: Vp0 = no PVTT, Vp1 = presence of a PVTT distal to, but not in, the second-order portal vein branches, Vp2 = presence of a PVTT in the second-order portal vein branches, Vp3 = presence of a PVTT in the first-order portal vein branches, and Vp4 = presence of a PVTT in the main trunk of the portal vein or a portal vein branch contralateral to the primarily involved lobe (or both)

### Liver function and Toxicity

A total of 9 patients (9/69, 13.0%) experienced an increase of 2 or more points in their Child–Pugh score at the 3-month follow-up (Table 4). Among these, 3 patients (3/9, 33.3%) remained in the same class, 5 patients (5/9, 55.6%) deteriorated from class A to B, and 1 patient (1/9, 11.1%) deteriorated from class B to C.

**Table 4:** Comparison of liver function parameters before and after radiation therapy

	**Pre-RT**	**3-month follow-up**	***p*-value**
Median CPS	5 (IQR, 5–6)	5 (IQR, 5–7)	*P* < 0.001
Patients with CPS Increase ≥ 2		13.0% (9/69)	
Median ALBI Score	-2.36 (IQR, -2.69– -1.90)	-2.18 (IQR, -2.73– -1.67)	*P* = 0.051
Patients with ALBI Grade Increase		21.7% (15/69)	

Abbreviations: *RT* Radiation therapy, *CPS* Child–Pugh score, *ALBI* Albumin-bilirubin, *IQR* Interquartile range

The median ALBI score before RT was -2.36 (IQR, -2.69 – -1.90) and at the 3-month follow-up was -2.18 (IQR, -2.73 – -1.67), with no significant difference between pre- and post-treatment (*p* = 0.051). Additionally, 21.7% of patients (15/69) experienced an increase in ALBI grade, with all cases showing a single-grade increase.

The treatment-related toxicities are presented in Table 5. During the 3 months after RT, almost all acute toxicities were of grade < 3, temporary, easily manageable, and did not cause any interruption in the treatment course. There were 2 (2.9%) cases of grade ≥ 3 toxicity: grade 3 liver enzyme elevation (*n* = 1), and acute cholangitis (*n* = 1). Both patients were admitted to the hospital, treated with conservative or antibacterial therapy, and completely cured. After 5 months following RT, late gastrointestinal toxicities were observed in 2 patients (2.9%), with grade 2 duodenal ulcers. These patients were successfully treated with endoscopic procedures and antacids, including proton pump inhibitors. No late hepatic failure or treatment-related death was observed.

**Table 5:** Treatment related acute and late toxicity

	**CTCAE grade**		
	**Grade 1**	**Grade 2**	**Grade 3**
**Acute toxicity**	n (%)	n (%)	n (%)
Fatigue	5 (7.2)	2 (2.9)	0
Anorexia	1 (1.4)	0	0
Nausea	6 (8.7)	5 (7.2)	0
Vomiting	3 (4.3)	0	0
Abdominal pain	1 (1.4)	1 (1.4)	0
Diarrhea	2 (2.9)	0	0
Thrombocytopenia	0	1 (1.4)	0
Cholangitis	0	0	1 (1.4)
AST / ALT elevation	0	0	1 (1.4)
**Late toxicity**			
Duodenal ulcer	0	2 (2.9)	0
**Total**	18 (26.1%)	11 (15.9%)	2 (2.8%)

Abbreviations: *AST* Aspartat aminotransferase, *ALT* Alanin aminotransferase

## Discussion

For patients with HCC and PVTT, maintaining liver function is crucial, as it directly impacts treatment options and overall outcomes. PVTT exacerbates liver dysfunction by reducing portal blood flow and contributing to portal hypertension, further complicating disease management [20]. In addition, intrahepatic metastasis and extrahepatic spread often coexist, creating additional challenges for treatment planning. Effective control of intrahepatic lesions is therefore important, as it has been shown to improve survival outcomes. For instance, the TACTICS trial demonstrated that combining TACE with sorafenib significantly prolonged PFS and delayed intrahepatic tumor progression, leading to improved overall survival [33]. Similarly, a randomized controlled trial conducted by Yoon et al. [14] highlighted the survival benefit of combining TACE and RT, compared to sorafenib alone, in controlling intrahepatic disease.

This study aimed to evaluate the role of RT as a bridging therapy before initiating systemic treatments. Our study demonstrated a 1-year OS of 62.2% and a median survival of 20.3 months. These results are comparable to those reported in recent systemic therapy trials. For example, the IMBRAVE150 post hoc analysis [12] demonstrated a median OS of 17.0 months with the atezolizumab-bevacizumab combination, while Kudo et al. [34] reported a median OS of 13.6 months with lenvatinib in advanced HCC. These comparisons suggest that 10-fraction hypofractionated IGRT offers competitive survival outcomes with relatively low toxicity. Based on our results, RT appears to be a promising bridging therapy for PVTT, preserving the potential for subsequent systemic treatments while providing effective disease control.

In this study, our hypofractionated regimen for PVTT-targeted IGRT showed a 1-year FFLP of 88.7%, which was consistent with local control rates reported in previous studies, ranging from 75 to 100 [14, 20, 29, 35–37]. The 1-year PFS and DMFS were 26.9% and 64.7%, respectively. Although these treatment outcomes showed similar results compared to previous studies, the absolute value of 1-year PFS appeared to be relatively low, with a total of 52 out of 69 patients experiencing out-field intrahepatic progression during follow-up. Chronic inflammation of the liver arising from chronic liver disease and intrahepatic hematogenous tumor spread seem to be associated with the low PFS rate [38].

An analysis of in-field recurrences revealed that tumor regrowth is commonly presented as diffuse enlargement rather than localized progression in specific regions. This recurrence pattern suggests that the prescribed radiation dose may have been globally insufficient to achieve complete tumor control, rather than inadequate delivery to specific subregions of the PVTT. As highlighted by Kim et al. [39], higher BEDs are associated with improved local control rates in HCC. However, in our study, a dose of 50 Gy administered in 10 fractions (BED: 75Gy_10_) was chosen to balance tumor control with the need to protect OARs. Escalating the dose beyond this level was considered unfeasible due to the increased risk of exacerbating liver dysfunction and potential toxicities to adjacent critical structures such as the biliary tree or gastrointestinal organs.

Several studies have indicated that higher radiation doses may enhance radiologic tumor response and overall survival [15, 16, 29, 30, 36, 40, 41]. Kim et al. [15] and Kim et al. [41] found that HCC with PVTT patients showed a better response rate (50–54.6%) with higher BED (58-64Gy_10_), and RT responders lived longer than non-responders (median survival 10.7 vs 5.3 months, 20.1 vs 7.2 months, respectively). Additionally, the studies of Khorprasert et al. [29] and Toya et al. [16] observed an improved response rate (80–82.8%) and survival outcome (1-year OS 59.3%, median survival 14.4 months, respectively) with higher BED (56-58Gy_10_). In our study, the median BED was 75Gy_10_ (56-75Gy_10_), which could be classified as a "high" BED according to the above studies. However, we found no significant difference in OS based on the 3-month response (HR 0.86, 95% CI 0.42–1.78, *p* = 0.88) in multivariate analysis.

The dose–response relationship in HCC treatment remains a subject of ongoing investigation. While higher radiation doses are generally associated with improved local control and survival outcomes, this relationship appears to be less well-defined for HCC than for liver metastases [23]. Recent studies, including those by the ISRS consortium [24] and Ohri N et al. [23], have highlighted that despite the traditional association between higher doses and improved outcomes, an optimal dosing strategy for HCC has not yet been definitively established. A key factor contributing to this uncertainty is the potential adverse effect of RT on cirrhotic patients. Liver decompensation, a common complication following RT, is particularly prevalent in individuals with pre-existing cirrhosis or impaired liver function [42]. These findings highlight the need for further research to identify dosing regimens that can achieve maximal therapeutic efficacy while minimizing the risk of liver-related complications.

Several studies have reported notable survival and local control rates using advanced radiation techniques such as PBT [20, 21] or SBRT [18, 19, 37, 40, 43] for patients with HCC and PVTT. In sequential phase I and II trials conducted by Bujold et al. [18], 54.9% of patients had tumor vascular thrombosis, and were treated with 24-54 Gy in 6 fractions. The 1-year local control rate was 87%, and the median survival was 17 months. However, there were 7 treatment-related deaths, including 5 cases of liver failure, among 102 patients, despite the mean liver dose being 18.1 Gy. In a recent retrospective analysis of SBRT (32-50 Gy in 5–6 fractions), a 1-year local control rate of 95% and a median survival of 15 months were reported, but 13 cases of grade ≥ 3 toxicity were observed among 29 patients [19].

On the other hand, PBT appears to offer distinct advantages in managing PVTT. Kim et al. [20] reported that PVTT patients treated with PBT (62.5–91.3GyE_10_ in 10 fractions) using the SIB technique achieved a 2-year local control rate of 88.1%, a 2-year OS of 51.1%, and a median survival of 34.4 months, with no cases of grade ≥ 3 toxicity or treatment-related hepatic failure. Similarly, a study conducted by Ishida et al. [21] reported a remarkable 5-year local control rate of 86.1%, with grade ≥ 3 toxicity observed only in 2.6% (3/116) of patients. The clinical outcomes of studies using various RT techniques for HCC with PVTT are summarized in Table 6.

**Table 6:** Clinical outcomes of studies using various RT techniques for HCC with PVTT

Reference​	No. of cases	Techniques​	Total dose​ (Gy)​	Fx size (Gy)​	Response rate (%) or 1-year local control (%)​	Median survival (months)​	Toxicity​ (CTCAE grade)
This study	69	IGRT (VMAT)	40–50	4–5	1-year local control 88.7%	18.5	Gr3 Cholangitis #1 Gr3 liver enzyme elevation #1 Gr2 Duodenal ulcer #2
Kim et al.​ 2005	59​	3D-CRT​	30–54​	2–3​	Response rate 55% with​ BED > 58Gy_10_​	Responder 10.7​ Non-responder 5.3​	Gr2 Gastrointesitnal ulcer #5 ​ RILD #1 ​
Toya et al.​ 2007	38​	3D-CRT​	17.5–50.4​	1.8–4​	Response rate 80% with​ BED > 58Gy_10_​	9.6​	Gr1-2 Duodenal ulcer #2
Bujold et al.​ 2013​	102 (n = 56 with PVTT)	SBRT	24–54	4–9	1-year local control 87%​	17.0	Gr5 Liver failure #5​ Gr5 Duodenal ulcer bleeding​ #1 Gr5 Cholangitis​ #1 Gr3-4 toxicity #30
Kumar et al.​ 2021​	29​	SBRT	32–50	8 (median)	1-year local control 95%	15.0	Gr 1 hepatic encephalopathy​ Gr 3 Lymphocytopenia #9​ Gr 3–4 Liver enzyme elevation ​#2​ Gr 3–4 Bilirubin level elevation ​#2​
Kim et al 2017	41	PBT	50–66 (RBE)	5–6.6 (RBE)	2-year local control 88.1%	34.4	Gr2 Gastrointestinal ulcer #2
Ishida et al 2024	116	PBT	72.6 (RBE)	3.3 (RBE)	1-year local control 95.1% 5-year local control 86.1%	11.0	Gr2 Gastrointestinal ulcer/stenosis #2 Gr3 Gastrointestinal ulcer/stenosis #2 Gr3 Cholecystitis #1

Abbreviations: *IGRT* Image-guided radiation therapy, *VMAT* Volumetric modulated arc therapy, *3D-CRT* Three-dimensional conformal radiation therapy; BED_10_, Biologically effective dose, as the α/β = 10; RILD, Radiation-induced liver disease; SBRT Stereotactic body radiotherapy; PBT, Proton beam therapy; RBE, Relative biological effectiveness

This study aimed to minimize setup uncertainties by utilizing IGRT for every fraction, ensuring precise treatment delivery. As a result, the 1-year FFLP rate was 88.7%, comparable to other studies on RT for PVTT. Notably, however, some studies on PBT have reported superior outcomes [20, 21]. This disparity might be attributed to the unique advantages of PBT, including its ability to spare OARs such as the liver, small bowel, and duodenum, as well as the use of high relative biological effectiveness (RBE) beams. Consequently, PBT may offer a valuable alternative for managing extensive PVTT, potentially reducing liver toxicity while achieving better local control. Nonetheless, PBT has limitations; it cannot completely avoid critical structures such as the biliary tree, which is anatomically parallel to the portal vein. This proximity leaves patients at risk of complications, such as biliary strictures.

By risk group, which was determined through multivariate analysis of our cohort, the 1-year OS was significantly lower in the high-risk group (43% vs 77%, *p* < 0.01). Risk factors in this high-risk group included high mUICC stage (IVA or IVB), Child–Pugh class B or C, and pre-treatment AFP ≥ 600 IU/ml. These factors align with risk factors suggested in scoring systems from previous studies, where ECOG ≥ 2, Child–Pugh class B or C, elevated AFP, large tumor size (≥ 5 cm or ≥ 10 cm), multiple tumors (> 2), main or bilateral portal vein involvement, complete portal vein occlusion, and lymph node or extrahepatic metastasis were identified as prognostic factors for OS of PVTT patients [44–46].

As our study found that low-risk group patients who received RT had better OS, while RT was not as effective in high-risk group patients, the scoring system used in this study could aid in determining the optimal treatment option, whether it be RT or systemic therapy, for patients with PVTT. However, further external validation is necessary.

There were only 2 cases of grade ≥ 3 toxicity and no treatment-related deaths or liver failure. Although the prescribed dose (BED: 56-75Gy_10_) was lower than in SBRT studies, the radiation dose could be considered "high," and the SIB technique with every fraction of IGRT might have reduced radiation toxicity to OARs. A case of grade 3 cholangitis occurred during treatment. The patient was hospitalized, treated with intravenous antibiotics and fully recovered. The other case of grade 3 toxicity was hepatic enzyme elevation, which was detected during out-patient follow-up, and the patient fully recovered with hospitalization and conservative therapy.

This study has inherent limitations, like other single-institution retrospective analyses. Although all patients received TACE prior to RT, the treatment interval between the two was variable. Considering that TACE followed by RT within 3 weeks showed a longer overall survival rate and higher response rate in a randomized clinical trial [14], the TACE-RT interval could have influenced the treatment outcomes in our study. Additionally, the radiologic response of PVTT was challenging to assess, and the treatment outcomes might have been affected by subsequent interventions following RT. Furthermore, due to the lack of robustness analysis based on 4DCT data, the uncertainties related to respiratory motion and other factors cannot be fully assessed in our study.

## Conclusions

Hypofractionated IGRT using the SIB technique demonstrated promising outcomes in terms of local control and overall survival for patients with PVTT, with acceptable toxicity. A 10-fraction image-guided hypofractionated RT regimen (BED: 56–75 Gy_10)_ achieved treatment results comparable to those of advanced RT modalities such as SBRT or PBT while also showing potential as a bridging therapy to systemic therapy. Additionally, our scoring system could help in predicting prognosis and selecting the optimal treatment option for PVTT patients, although further validation is needed.

## Data Availability

The datasets used and/or analyzed during the current study are available from the corresponding author on reasonable request.

## Abbreviations

AFP: Alpha-fetoprotein
BCLC: Barcelona Clinic for Liver Cancer
BED: Biologically effective dose
CI: Confidence interval
CR: Complete response
CT: Computed tomography
CTCAE: National Cancer Institute Common Terminology Criteria for Adverse Events
DMFS: Distant metastasis-free survival
DVH: Dose-volume histogram
ECOG: Eastern Cooperative Oncology Group
FFLP: Freedom from local progression
GTV: Gross tumor volume
HCC: Hepatocellular carcinoma
HR: Hazard ratio
IGRT: Image-guided radiation therapy
IQR: Interquartile range
ITV: Internal target volume
MRI: Magnetic resonance imaging
mUICC: Modified Union for International Cancer Control staging system
OAR: Organ-at-risk
OS: Overall survival
PBT: Proton beam therapy
PD: Progressive disease
PEIT: Percutaneous ethanol injection therapy
PFS: Progression-free survival
PIVKA-II: Protein induced by vitamin K absence-II
PR: Partial response
PTV: Planning target volume
PVTT: Portal vein tumor thrombosis
RECIST: Response Evaluation Criteria in Solid Tumors
RFA: Radiofrequency ablation
RT: Radiation therapy
SBRT: Stereotactic body radiotherapy
SD: Stable disease
SIB: Simultaneous integrated boost
TACE: Transarterial chemoembolization
TARE: Transarterial radioembolization
VMAT: Volumetric-modulated radiotherapy
